# Crystal structure of aqua­tris­{μ-*N*-[bis(diethyl­amino)phosphoryl]-2,2,2-tri­chloroacetamidato-κ^3^ *O*,*O*′:*O*}calciumsodium

**DOI:** 10.1107/S2056989016017035

**Published:** 2016-11-01

**Authors:** Iuliia Shatrava, Kateryna Gubina, Vladimir Ovchynnikov, Viktoriya Dyakonenko, Vladimir Amirkhanov

**Affiliations:** a Taras Shevchenko National University of Kyiv, Department of Chemistry, Volodymyrska str., 64, 01033 Kyiv, Ukraine; b STC "Institute for Single Crystals", 60 Nauki ave., Kharkiv 61072, Ukraine

**Keywords:** crystal structure, carbacyl­amido­phosphates, calcium sodium binuclear compounds

## Abstract

In the mol­ecular structure of the title compound, [CaNa(C_10_H_20_Cl_3_N_3_O_2_P)_3_(H_2_O)], the Ca^2+^ ion has a slightly distorted octa­hedral coordination environment defined by six O atoms which belong to the carbonyl and phosphoryl groups of the three coordinating ligands. Two Cl atoms of CCl_3_ groups and four O atoms form the coordination environment of the Na^+^ ion: three from the carbonyl groups of ligands and one O atom from a coordinating water mol­ecule. In the crystal, the bimetallic complexes are assembled into chains along the *c*-axis direction *via* O—H⋯O hydrogen bonds that involve the coordinating water mol­ecules and the phosphoryl groups.

## Abstract 2

In the title compound, three ligands are coordinated to the Ca^2+^ ion in a bidentate-chelated manner *via* the O atoms of the CO and PO groups. The sodium ion is encapsulated between three chelated metallacycles {Ca*L*}(where *L* ^−^ = Cl_3_CC(O)NP(O)[N(C_2_H_5_)_2_]_2_) due to the bridging function of the carbonyl O atoms and it is capped by the water mol­ecule.

## Chemical context   

In recent years, the inter­est of many researchers has been focused on metal–phospho­rus containing chelates and their usefulness as reagents (principally the alkali metal derivatives) and as potential precursors (the alkaline earth derivatives) for chemical vapor deposition (CVD) (Hanusa, 2003 ▸), thin films (Hitzbleck *et al.*, 2004 ▸; Demadis *et al.*, 2009 ▸, 2010 ▸), anti­tumor activity (Liu *et al.*, 2012 ▸) and as models for calcium-binding proteins (bearing biologically relevant ligands) (Hoang *et al.*, 2003 ▸).

Polyfunctional phospho­rus compounds [O=P—C(*R*)—P=O](*L*), having oxygen-donor groups capable of binding a number of metal ions into structurally versatile metal phospho­nate hybrids *M* ^2+^/*L* (Sr^2+^, Ba^2+^, Ca^2+^) or *A* ^+^/*M* ^2+^/*L* (*A* = Na, K) have received considerable attention (Colodrero *et al.*, 2011 ▸; Niekiel & Stock, 2014 ▸). Complexes based on carbacyl­amido-­phosphates (CAPhos) containing the phospho­rylated structural core [O=C—NH—P=O] have been used as luminescence markers (Litsis *et al.*, 2015 ▸), for their cytotoxic activity (Grynuyk *et al.*, 2016 ▸) and as building-blocks in aimed synthesis of coordination compounds with specified structure (Shatrava *et al.*, 2016 ▸). The especially inter­esting feature of carbacyl­amido­phosphate ligands is the bidentate or bidentate–chelate character of their coordination to the central atom (Amirkhanov *et al.*, 2014 ▸; Gubina *et al.*, 2000 ▸). On this subject, two papers related to complexes of an alkali element in the coordination chemistry of carbacyl­amido­phosphates have been published (Trush *et al.*, 2005 ▸; Litsis *et al.*, 2010 ▸).

The present paper is devoted to the synthesis and structural analysis of a Ca^2+^-containing complex [CaNa(*L*)_3_(H_2_O)], (I), in which the Na^+^ ion is four-coordinate and has additional contacts with two Cl atoms and where *L* ^−^ is the CAPhos ligand with a bidentate–chelate and bridging function of the carbonyl group.
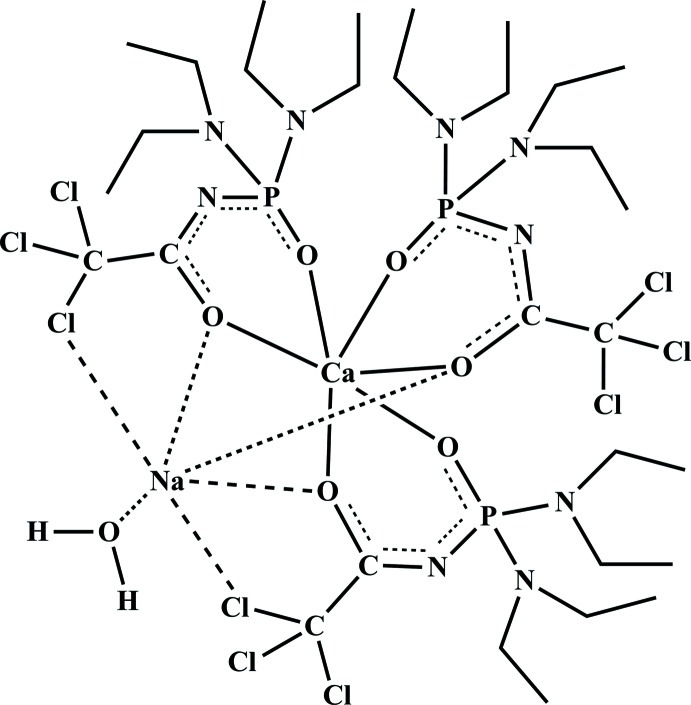


## Structural commentary   

**Figure 1:**
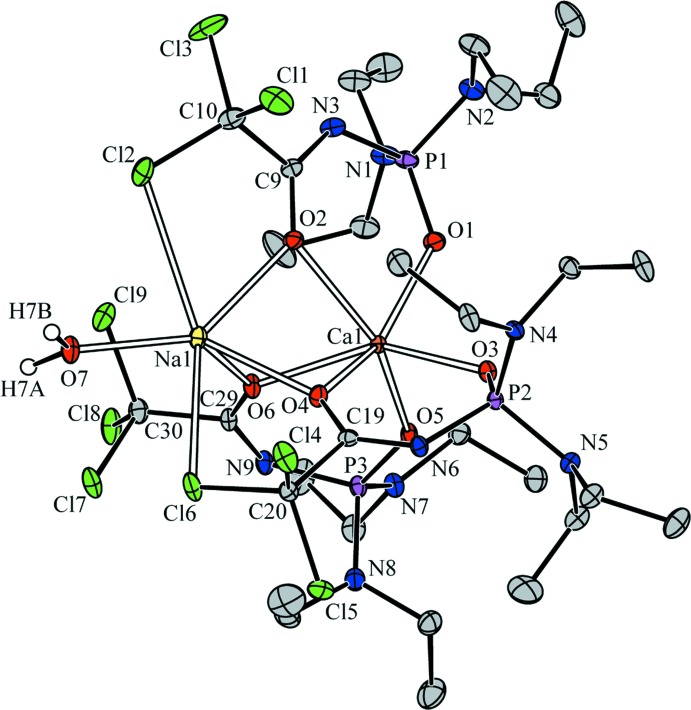
The mol­ecular structure of (I), showing 30% probability displacement ellipsoids and the atom-numbering scheme. Labels and H atoms of ethyl groups have been omitted for clarity.

In the title structure (Fig. 1 ▸), the Ca atom is coordinated by all six O atoms of three bidentate chelating CAPhos ligands in a distorted octa­hedral geometry. The Ca—O(C) bond lengths [2.371 (2)–2.392 (2) Å] are longer than the Ca—O(P) bonds [2.262 (2)–2.323 (2) Å]. Similar Ca—O(P) bond lengths of 2.283 (6)–2.332 (6) Å are found in the structures of [Ca{Ph_2_P(O)CH_2_P(O)Ph_2_}_3_]^2+^ (Hursthouse *et al.*, 2005 ▸) and [Ca(C_8_H_11_NO_5_PS)_2_]_*n*_ (Trush *et al.*, 2009 ▸).

The P=O, C—N and C=O bond lengths in (I) are in good agreement with those observed for complexes based on CAPhos ligands (Amirkhanov *et al.*, 2014 ▸). The coordination polyhedron around Na^+^ has a distorted tetra­hedron-like geometry, formed by three carbonyl oxygen atoms from three ligands and one from the coordinating water mol­ecule with O(C)—Na—O(C) and O(C)—Na—O(W) angle ranges of 76.19 (8)–77.48 (7)° and 126.09 (10)–141.26 (9)°, respectively. The Na ion also has additional contacts with two Cl atoms of CCl_3_ groups [2.976 (1) and 3.086 (1) Å. The Na—O(W) bond length [2.276 (2) Å] is significantly shorter than the Na—O(C) bonds [2.333 (2)–2.393 (2) Å]. A similar type of bonding was observed earlier in [Na_2_(C_10_H_16_Cl_3_N_3_O_4_P)_2_(H_2_O)_2_]_*n*_ (Litsis *et al.*, 2010 ▸), [Na{Ph_2_P(O)CH_2_P(O)Ph_2_}_3_Cl] (Ding *et al.*, 2000 ▸), [NaNd(C_14_H_21_N_3_O_5_PS)_4_]_*n*_ (Shatrava *et al.*, 2010 ▸) and [NaNd(C_8_H_11_NO_5_PS)_4_]_*n*_ (Moroz *et al.*, 2007 ▸). The Ca⋯Na distance of 3.321 (3) Å is much shorter than that in [CaNa(PC)_2_(H_2_O)]_*n*_ [4.3972 (5) Å; PC = phospho­citrate ligand; Demadis, 2003 ▸).

## Supra­molecular features   

**Figure 2:**
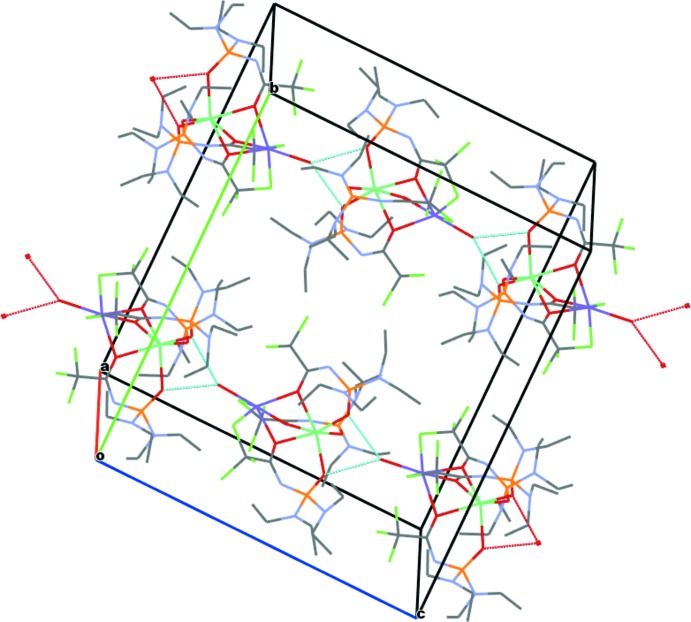
The mol­ecular packing for (I), showing hydrogen-bonded chains running along the *c* axis. O—H⋯O hydrogen bonds are shown as dashed lines.

In the crystal, the complex mol­ecules are linked into chains along the *c* axis *via* O—H⋯O hydrogen-bonding inter­actions (Fig. 2 ▸, Table 1 ▸) in which the water O atom acts as a donor, and the O atoms of the two phosphoryl groups of a neighbouring mol­ecule act as the acceptors.

## Database survey   

A search of the Cambridge Structural Database (Version 5.37, with one update; Groom *et al.*, 2016 ▸) returned five entries for crystal structures of calcium sodium binuclear compounds with phospho­rus-containing acids (Demadis *et al.*, 2001 ▸). Only one binuclear coordination compound based on the CAPhos ligand with an encapsulated sodium cation is known, *viz*. NaEr*L*_4_·H_2_O (Amirkhanov *et al.*, 1996*a* ▸).

## Synthesis and crystallization   

The synthesis of H*L* was carried out according to a previously reported method (Amirkhanov *et al.*, 1996*b* ▸). Anhydrous CaCl_2_ (0.027 g, 0.24 mmol) was dissolved in hot methanol and added to a solution of Na*L* (0.257 g, 0.73 mmol) in acetone. Colorless crystals of the complex suitable for X-ray diffraction could be separated over a period of three days; they were washed with acetone. IR (KBr pellet, cm^−1^): 1618 (*s*, CO) and 1110 (*s*, PO).

## Refinement   

Crystal data, data collection and structure refinement details are summarized in Table 2 ▸. All C-bound H atoms were idealized (C–H = 0.98–0.99 Å) and refined within the riding-model approximation with *U*_iso_(H) = 1.2 or 1.5 *U*_eq_(C). The coordinates of water H atoms were freely refined, with *U*_iso_(H) = 1.5*U*_eq_(O).

## Supplementary Material

Crystal structure: contains datablock(s) I. DOI: 10.1107/S2056989016017035/bg2595sup1.cif

Structure factors: contains datablock(s) I. DOI: 10.1107/S2056989016017035/bg2595Isup2.hkl

CCDC reference: 1511311

Additional supporting information: crystallographic information; 3D view; checkCIF report

## Figures and Tables

**Table 1:** Hydrogen-bond geometry (Å, °)

*D*—H⋯*A*	*D*—H	H⋯*A*	*D*⋯*A*	*D*—H⋯*A*
O7—H7*A*⋯O3^i^	0.86 (4)	2.23 (4)	2.959 (3)	143 (3)
O7—H7*B*⋯O5^i^	0.80 (4)	2.08 (4)	2.843 (3)	159 (4)

Symmetry code: (i) 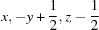.

**Table 2:** Experimental details

Crystal data	
Chemical formula	[CaNa(C_10_H_20_Cl_3_N_3_O_2_P)_3_(H_2_O)]
*M*_r_	1135.91
Crystal system, space group	Monoclinic, *P*2_1_/*c*
Temperature (K)	100
*a*, *b*, *c* (Å)	13.4014 (5), 21.8127 (10), 18.2427 (6)
β (°)	100.539 (4)
*V* (Å^3^)	5242.8 (4)
*Z*	4
Radiation type	Mo *K*α
μ (mm^−1^)	0.73
Crystal size (mm)	0.5 × 0.3 × 0.2

Data collection	
Diffractometer	Agilent Xcalibur, Sapphire3
Absorption correction	Multi-scan (*CrysAlis PRO*; Agilent, 2013 ▸)
*T*_min_, *T*_max_	0.981, 1.000
No. of measured, independent and observed [*I* > 2σ(*I*)] reflections	56299, 16965, 10752
*R*_int_	0.077
(sin θ/λ)_max_ (Å^−1^)	0.757

Refinement	
*R*[*F* ^2^ > 2σ(*F* ^2^)], *wR*(*F* ^2^), *S*	0.063, 0.131, 1.05
No. of reflections	16965
No. of parameters	559
H-atom treatment	H atoms treated by a mixture of independent and constrained refinement
Δρ_max_, Δρ_min_ (e Å^−3^)	0.81, −0.53

Computer programs: *CrysAlis PRO* (Agilent, 2013 ▸), *SHELXT* (Sheldrick, 2015*a* ▸), *SHELXL2014* (Sheldrick, 2015*b* ▸) and *OLEX2* (Dolomanov *et al.*, 2009 ▸).

## supplementary crystallographic information

### Crystal data

**Table:**

[CaNa(C_10_H_20_Cl_3_N_3_O_2_P)_3_(H_2_O)]	*F*(000) = 2360
*M**_r_* = 1135.91	*D*_x_ = 1.439 Mg m^−^^3^
Monoclinic, *P*2_1_/*c*	Mo *K*α radiation, λ = 0.71073 Å
*a* = 13.4014 (5) Å	Cell parameters from 9777 reflections
*b* = 21.8127 (10) Å	θ = 2.9–31.1°
*c* = 18.2427 (6) Å	µ = 0.73 mm^−^^1^
β = 100.539 (4)°	*T* = 100 K
*V* = 5242.8 (4) Å^3^	Block, colourless
*Z* = 4	0.5 × 0.3 × 0.2 mm

### Data collection

**Table:**

Agilent Xcalibur, Sapphire3 diffractometer	16965 independent reflections
Radiation source: Enhance (Mo) X-ray Source	10752 reflections with *I* > 2σ(*I*)
Graphite monochromator	*R*_int_ = 0.077
Detector resolution: 16.1827 pixels mm^-1^	θ_max_ = 32.5°, θ_min_ = 2.9°
ω scans	*h* = −17→19
Absorption correction: multi-scan (CrysAlis PRO; Agilent, 2013)	*k* = −32→32
*T*_min_ = 0.981, *T*_max_ = 1.000	*l* = −21→26
56299 measured reflections	

### Refinement

**Table:**

Refinement on *F*^2^	Primary atom site location: structure-invariant direct methods
Least-squares matrix: full	Hydrogen site location: mixed
*R*[*F*^2^ > 2σ(*F*^2^)] = 0.063	H atoms treated by a mixture of independent and constrained refinement
*wR*(*F*^2^) = 0.131	*w* = 1/[σ^2^(*F*_o_^2^) + (0.0333*P*)^2^ + 3.9976*P*] where *P* = (*F*_o_^2^ + 2*F*_c_^2^)/3
*S* = 1.05	(Δ/σ)_max_ = 0.001
16965 reflections	Δρ_max_ = 0.81 e Å^−^^3^
559 parameters	Δρ_min_ = −0.53 e Å^−^^3^
0 restraints	

### Special details

**Table:**

Geometry. All esds (except the esd in the dihedral angle between two l.s. planes) are estimated using the full covariance matrix. The cell esds are taken into account individually in the estimation of esds in distances, angles and torsion angles; correlations between esds in cell parameters are only used when they are defined by crystal symmetry. An approximate (isotropic) treatment of cell esds is used for estimating esds involving l.s. planes.

### Fractional atomic coordinates and isotropic or equivalent isotropic displacement parameters (Å^2^)

**Table:**

	*x*	*y*	*z*	*U*_iso_*/*U*_eq_	
Ca1	0.30938 (4)	0.26175 (3)	0.05317 (3)	0.01394 (11)	
Cl1	0.52209 (7)	0.41961 (4)	−0.07288 (5)	0.0412 (2)	
Cl2	0.49248 (6)	0.31147 (4)	−0.16544 (4)	0.03247 (19)	
Cl3	0.68577 (7)	0.33489 (6)	−0.07300 (6)	0.0556 (3)	
Cl4	0.02206 (7)	0.41154 (4)	−0.15774 (4)	0.03229 (19)	
Cl5	−0.08582 (6)	0.31288 (5)	−0.10258 (4)	0.0352 (2)	
Cl6	0.06795 (6)	0.28761 (4)	−0.18889 (4)	0.02943 (18)	
Cl7	0.20528 (7)	0.10653 (4)	−0.18555 (4)	0.03215 (19)	
Cl8	0.35228 (7)	0.02340 (4)	−0.10288 (4)	0.0350 (2)	
Cl9	0.41532 (7)	0.14074 (4)	−0.14729 (4)	0.0340 (2)	
P1	0.56560 (6)	0.27334 (4)	0.12621 (4)	0.02079 (17)	
P2	0.15063 (5)	0.37823 (3)	0.08914 (4)	0.01445 (14)	
P3	0.22119 (6)	0.11337 (4)	0.07838 (4)	0.01754 (15)	
Na1	0.29650 (9)	0.26717 (6)	−0.13032 (6)	0.0213 (3)	
O1	0.45756 (15)	0.26654 (11)	0.13545 (10)	0.0231 (5)	
O2	0.42012 (14)	0.29851 (10)	−0.02518 (10)	0.0212 (5)	
O3	0.22811 (14)	0.32988 (9)	0.11674 (10)	0.0174 (4)	
O4	0.19420 (14)	0.30713 (10)	−0.04789 (10)	0.0198 (4)	
O5	0.25053 (16)	0.17625 (10)	0.10821 (10)	0.0200 (4)	
O6	0.29464 (16)	0.18838 (10)	−0.04410 (10)	0.0212 (4)	
O7	0.27508 (19)	0.25628 (12)	−0.25633 (11)	0.0259 (5)	
H7A	0.275 (3)	0.221 (2)	−0.276 (2)	0.039*	
H7B	0.267 (3)	0.2825 (19)	−0.287 (2)	0.039*	
N1	0.62190 (18)	0.20676 (13)	0.14390 (14)	0.0249 (6)	
N2	0.63464 (19)	0.32149 (13)	0.18381 (13)	0.0240 (6)	
N3	0.58423 (18)	0.29959 (13)	0.04559 (13)	0.0229 (6)	
N4	0.19814 (18)	0.44687 (12)	0.08862 (12)	0.0180 (5)	
N5	0.06822 (18)	0.37906 (11)	0.14712 (12)	0.0173 (5)	
N6	0.08462 (17)	0.36888 (11)	0.00484 (12)	0.0176 (5)	
N7	0.26564 (19)	0.06364 (12)	0.14352 (13)	0.0215 (5)	
N8	0.09859 (19)	0.10053 (13)	0.05679 (13)	0.0238 (6)	
N9	0.26156 (19)	0.09295 (12)	0.00233 (12)	0.0202 (5)	
C1	0.5617 (3)	0.15065 (16)	0.13558 (19)	0.0315 (8)	
H1A	0.4956	0.1592	0.1504	0.038*	
H1B	0.5970	0.1194	0.1703	0.038*	
C2	0.5423 (3)	0.1244 (2)	0.0581 (2)	0.0536 (12)	
H2A	0.5086	0.1551	0.0230	0.080*	
H2B	0.4988	0.0881	0.0567	0.080*	
H2C	0.6070	0.1127	0.0443	0.080*	
C3	0.7291 (2)	0.20065 (18)	0.1366 (2)	0.0338 (8)	
H3A	0.7618	0.2415	0.1417	0.041*	
H3B	0.7330	0.1844	0.0866	0.041*	
C4	0.7852 (3)	0.1579 (2)	0.1958 (2)	0.0478 (10)	
H4A	0.7630	0.1156	0.1843	0.072*	
H4B	0.7706	0.1695	0.2447	0.072*	
H4C	0.8584	0.1610	0.1967	0.072*	
C5	0.6416 (3)	0.31108 (19)	0.26430 (17)	0.0353 (8)	
H5A	0.6133	0.2702	0.2721	0.042*	
H5B	0.5999	0.3421	0.2844	0.042*	
C6	0.7500 (3)	0.3146 (2)	0.3069 (2)	0.0456 (10)	
H6A	0.7928	0.2864	0.2844	0.068*	
H6B	0.7517	0.3031	0.3590	0.068*	
H6C	0.7754	0.3565	0.3046	0.068*	
C7	0.6476 (3)	0.38515 (17)	0.1624 (2)	0.0332 (8)	
H7C	0.6696	0.3856	0.1135	0.040*	
H7D	0.7024	0.4039	0.1993	0.040*	
C8	0.5531 (3)	0.4239 (2)	0.1572 (2)	0.0519 (11)	
H8A	0.5009	0.4087	0.1165	0.078*	
H8B	0.5692	0.4666	0.1474	0.078*	
H8C	0.5280	0.4215	0.2043	0.078*	
C9	0.5147 (2)	0.30817 (14)	−0.01230 (15)	0.0180 (6)	
C10	0.5541 (2)	0.34089 (17)	−0.07783 (17)	0.0279 (7)	
C11	0.2773 (2)	0.46682 (15)	0.15059 (15)	0.0229 (6)	
H11A	0.3383	0.4789	0.1306	0.027*	
H11B	0.2962	0.4318	0.1848	0.027*	
C12	0.2451 (3)	0.52007 (16)	0.19431 (16)	0.0322 (8)	
H12A	0.2276	0.5552	0.1610	0.048*	
H12B	0.3011	0.5313	0.2346	0.048*	
H12C	0.1860	0.5081	0.2156	0.048*	
C13	0.1801 (3)	0.48730 (15)	0.02316 (15)	0.0269 (7)	
H13A	0.1814	0.5304	0.0402	0.032*	
H13B	0.1117	0.4789	−0.0060	0.032*	
C14	0.2579 (3)	0.4792 (2)	−0.02705 (18)	0.0441 (11)	
H14A	0.2445	0.5089	−0.0680	0.066*	
H14B	0.2533	0.4375	−0.0474	0.066*	
H14C	0.3261	0.4861	0.0019	0.066*	
C15	−0.0128 (2)	0.42512 (15)	0.13284 (16)	0.0236 (6)	
H15A	−0.0685	0.4096	0.0940	0.028*	
H15B	0.0140	0.4630	0.1137	0.028*	
C16	−0.0545 (3)	0.44007 (17)	0.20263 (19)	0.0352 (8)	
H16A	−0.1024	0.4743	0.1924	0.053*	
H16B	0.0015	0.4515	0.2428	0.053*	
H16C	−0.0895	0.4041	0.2178	0.053*	
C17	0.0369 (2)	0.31950 (15)	0.17294 (17)	0.0236 (6)	
H17A	0.0126	0.3258	0.2205	0.028*	
H17B	0.0971	0.2924	0.1834	0.028*	
C18	−0.0452 (3)	0.2872 (2)	0.1191 (2)	0.0502 (11)	
H18A	−0.0212	0.2794	0.0723	0.075*	
H18B	−0.1059	0.3131	0.1092	0.075*	
H18C	−0.0616	0.2482	0.1408	0.075*	
C19	0.1140 (2)	0.33714 (13)	−0.04671 (14)	0.0154 (5)	
C20	0.0332 (2)	0.33663 (14)	−0.12081 (15)	0.0189 (6)	
C21	0.0375 (2)	0.10914 (17)	0.11532 (18)	0.0296 (7)	
H21A	−0.0120	0.1425	0.1002	0.036*	
H21B	0.0827	0.1219	0.1619	0.036*	
C22	−0.0188 (3)	0.05222 (19)	0.1303 (2)	0.0439 (10)	
H22A	−0.0653	0.0400	0.0848	0.066*	
H22B	−0.0575	0.0605	0.1699	0.066*	
H22C	0.0298	0.0191	0.1460	0.066*	
C23	0.0422 (3)	0.09849 (18)	−0.02034 (18)	0.0335 (8)	
H23A	−0.0205	0.0742	−0.0216	0.040*	
H23B	0.0840	0.0772	−0.0519	0.040*	
C24	0.0138 (3)	0.1614 (2)	−0.0531 (2)	0.0544 (12)	
H24A	−0.0256	0.1569	−0.1036	0.082*	
H24B	0.0757	0.1848	−0.0550	0.082*	
H24C	−0.0269	0.1831	−0.0218	0.082*	
C25	0.3321 (2)	0.08055 (16)	0.21407 (15)	0.0242 (7)	
H25A	0.3591	0.1223	0.2094	0.029*	
H25B	0.3904	0.0519	0.2236	0.029*	
C26	0.2770 (3)	0.0788 (2)	0.27963 (17)	0.0370 (9)	
H26A	0.2224	0.1093	0.2722	0.055*	
H26B	0.3249	0.0881	0.3256	0.055*	
H26C	0.2481	0.0379	0.2834	0.055*	
C27	0.2515 (3)	−0.00186 (16)	0.12843 (17)	0.0288 (7)	
H27A	0.1929	−0.0075	0.0873	0.035*	
H27B	0.2349	−0.0221	0.1732	0.035*	
C28	0.3436 (3)	−0.03324 (19)	0.1075 (2)	0.0425 (10)	
H28A	0.3598	−0.0142	0.0625	0.064*	
H28B	0.3288	−0.0768	0.0981	0.064*	
H28C	0.4016	−0.0290	0.1486	0.064*	
C29	0.2896 (2)	0.13098 (14)	−0.04402 (14)	0.0175 (6)	
C30	0.3164 (2)	0.10037 (15)	−0.11537 (15)	0.0236 (6)	

### Atomic displacement parameters (Å^2^)

**Table:**

	*U*^11^	*U*^22^	*U*^33^	*U*^12^	*U*^13^	*U*^23^
Ca1	0.0156 (3)	0.0131 (3)	0.0133 (2)	0.0009 (2)	0.0028 (2)	0.00007 (19)
Cl1	0.0513 (6)	0.0240 (5)	0.0446 (5)	−0.0105 (4)	−0.0008 (4)	0.0118 (4)
Cl2	0.0381 (5)	0.0402 (5)	0.0217 (3)	0.0048 (4)	0.0126 (3)	0.0056 (3)
Cl3	0.0214 (4)	0.0944 (10)	0.0543 (6)	0.0021 (5)	0.0153 (4)	0.0331 (6)
Cl4	0.0469 (5)	0.0223 (4)	0.0233 (4)	0.0071 (4)	−0.0051 (3)	0.0042 (3)
Cl5	0.0202 (4)	0.0513 (6)	0.0325 (4)	−0.0092 (4)	0.0006 (3)	−0.0041 (4)
Cl6	0.0339 (4)	0.0317 (5)	0.0198 (3)	0.0090 (4)	−0.0027 (3)	−0.0102 (3)
Cl7	0.0412 (5)	0.0340 (5)	0.0178 (3)	0.0065 (4)	−0.0036 (3)	−0.0068 (3)
Cl8	0.0609 (6)	0.0204 (4)	0.0253 (4)	0.0159 (4)	0.0120 (4)	−0.0013 (3)
Cl9	0.0403 (5)	0.0352 (5)	0.0315 (4)	0.0041 (4)	0.0197 (4)	−0.0002 (3)
P1	0.0164 (4)	0.0226 (4)	0.0215 (4)	−0.0025 (3)	−0.0015 (3)	0.0061 (3)
P2	0.0175 (3)	0.0124 (4)	0.0134 (3)	0.0015 (3)	0.0027 (3)	−0.0006 (3)
P3	0.0245 (4)	0.0132 (4)	0.0152 (3)	0.0002 (3)	0.0041 (3)	−0.0005 (3)
Na1	0.0260 (6)	0.0247 (7)	0.0135 (5)	0.0025 (5)	0.0043 (5)	0.0013 (4)
O1	0.0187 (10)	0.0335 (14)	0.0160 (9)	−0.0030 (9)	0.0005 (8)	0.0050 (9)
O2	0.0177 (10)	0.0281 (13)	0.0177 (9)	−0.0021 (9)	0.0031 (8)	0.0040 (8)
O3	0.0207 (10)	0.0152 (11)	0.0157 (9)	0.0031 (8)	0.0017 (8)	−0.0012 (7)
O4	0.0191 (10)	0.0241 (12)	0.0164 (9)	0.0063 (9)	0.0031 (8)	0.0000 (8)
O5	0.0321 (11)	0.0156 (11)	0.0136 (9)	−0.0030 (9)	0.0077 (8)	0.0000 (7)
O6	0.0324 (12)	0.0160 (11)	0.0162 (9)	0.0004 (9)	0.0071 (8)	−0.0002 (8)
O7	0.0426 (13)	0.0204 (13)	0.0155 (10)	0.0013 (11)	0.0071 (10)	0.0008 (8)
N1	0.0179 (12)	0.0221 (15)	0.0327 (14)	−0.0020 (11)	−0.0012 (11)	0.0089 (11)
N2	0.0235 (13)	0.0224 (15)	0.0240 (12)	−0.0038 (11)	−0.0010 (10)	0.0051 (10)
N3	0.0176 (12)	0.0241 (15)	0.0261 (12)	0.0016 (11)	0.0018 (10)	0.0077 (11)
N4	0.0205 (12)	0.0180 (13)	0.0148 (10)	−0.0036 (10)	0.0015 (9)	0.0003 (9)
N5	0.0214 (12)	0.0136 (13)	0.0183 (11)	0.0018 (10)	0.0069 (9)	0.0019 (9)
N6	0.0193 (12)	0.0161 (13)	0.0171 (11)	0.0032 (10)	0.0025 (9)	−0.0025 (9)
N7	0.0283 (13)	0.0173 (14)	0.0179 (11)	0.0008 (11)	0.0018 (10)	0.0013 (9)
N8	0.0245 (13)	0.0261 (15)	0.0210 (12)	0.0011 (11)	0.0051 (10)	−0.0022 (10)
N9	0.0286 (13)	0.0149 (13)	0.0173 (11)	0.0011 (11)	0.0052 (10)	−0.0011 (9)
C1	0.0333 (18)	0.0201 (18)	0.0408 (19)	−0.0048 (15)	0.0056 (15)	0.0042 (14)
C2	0.058 (3)	0.033 (2)	0.058 (3)	0.010 (2)	−0.019 (2)	−0.0165 (19)
C3	0.0220 (16)	0.029 (2)	0.048 (2)	−0.0003 (14)	0.0000 (15)	0.0085 (16)
C4	0.037 (2)	0.043 (3)	0.061 (3)	0.0092 (19)	0.0040 (19)	0.008 (2)
C5	0.041 (2)	0.037 (2)	0.0256 (16)	−0.0128 (17)	−0.0001 (15)	0.0051 (14)
C6	0.051 (2)	0.044 (3)	0.0330 (19)	0.012 (2)	−0.0152 (18)	−0.0026 (17)
C7	0.0310 (18)	0.0249 (19)	0.0400 (19)	−0.0062 (15)	−0.0036 (15)	0.0051 (15)
C8	0.054 (3)	0.033 (2)	0.063 (3)	0.015 (2)	−0.006 (2)	−0.003 (2)
C9	0.0178 (14)	0.0167 (15)	0.0204 (13)	0.0003 (11)	0.0057 (11)	0.0047 (11)
C10	0.0217 (15)	0.034 (2)	0.0282 (16)	−0.0006 (14)	0.0063 (13)	0.0110 (14)
C11	0.0247 (15)	0.0238 (18)	0.0186 (13)	−0.0040 (13)	−0.0001 (12)	0.0011 (12)
C12	0.053 (2)	0.0215 (18)	0.0192 (14)	−0.0015 (16)	−0.0015 (15)	−0.0048 (12)
C13	0.048 (2)	0.0149 (16)	0.0162 (13)	−0.0053 (14)	0.0013 (13)	0.0017 (11)
C14	0.062 (3)	0.049 (3)	0.0233 (16)	−0.028 (2)	0.0128 (17)	0.0003 (16)
C15	0.0255 (15)	0.0189 (17)	0.0290 (15)	0.0074 (13)	0.0117 (13)	0.0031 (12)
C16	0.043 (2)	0.025 (2)	0.045 (2)	0.0116 (16)	0.0254 (17)	0.0031 (15)
C17	0.0270 (16)	0.0167 (16)	0.0297 (15)	0.0052 (13)	0.0121 (13)	0.0061 (12)
C18	0.060 (3)	0.035 (2)	0.054 (2)	−0.027 (2)	0.008 (2)	0.0079 (19)
C19	0.0156 (13)	0.0146 (14)	0.0155 (12)	−0.0004 (11)	0.0012 (10)	0.0021 (10)
C20	0.0196 (14)	0.0167 (15)	0.0193 (13)	0.0013 (12)	0.0009 (11)	−0.0021 (11)
C21	0.0262 (16)	0.033 (2)	0.0306 (16)	0.0042 (15)	0.0081 (14)	−0.0020 (14)
C22	0.055 (2)	0.039 (3)	0.043 (2)	−0.004 (2)	0.0239 (19)	0.0048 (17)
C23	0.0292 (17)	0.039 (2)	0.0299 (17)	−0.0060 (16)	−0.0014 (14)	−0.0014 (15)
C24	0.044 (2)	0.065 (3)	0.051 (2)	0.012 (2)	−0.002 (2)	0.023 (2)
C25	0.0248 (15)	0.0296 (19)	0.0183 (13)	−0.0016 (14)	0.0043 (12)	0.0050 (12)
C26	0.038 (2)	0.053 (3)	0.0194 (15)	−0.0077 (19)	0.0047 (14)	0.0016 (15)
C27	0.0407 (19)	0.0199 (18)	0.0264 (15)	0.0014 (15)	0.0080 (14)	0.0037 (13)
C28	0.062 (3)	0.030 (2)	0.039 (2)	0.014 (2)	0.0179 (19)	0.0063 (16)
C29	0.0218 (14)	0.0160 (15)	0.0143 (12)	0.0031 (12)	0.0020 (11)	−0.0012 (10)
C30	0.0319 (17)	0.0197 (17)	0.0191 (13)	0.0048 (13)	0.0043 (12)	0.0015 (11)

### Geometric parameters (Å, º)

**Table:**

Ca1—O1	2.2621 (19)	C5—H5A	0.9900
Ca1—O2	2.380 (2)	C5—H5B	0.9900
Ca1—O3	2.281 (2)	C5—C6	1.519 (5)
Ca1—O4	2.3917 (19)	C6—H6A	0.9800
Ca1—O5	2.323 (2)	C6—H6B	0.9800
Ca1—O6	2.371 (2)	C6—H6C	0.9800
Cl1—C10	1.776 (4)	C7—H7C	0.9900
Cl2—C10	1.779 (3)	C7—H7D	0.9900
Cl3—C10	1.756 (3)	C7—C8	1.511 (5)
Cl4—C20	1.763 (3)	C8—H8A	0.9800
Cl5—C20	1.766 (3)	C8—H8B	0.9800
Cl6—C20	1.765 (3)	C8—H8C	0.9800
Cl7—C30	1.783 (3)	C9—C10	1.564 (4)
Cl8—C30	1.750 (3)	C11—H11A	0.9900
Cl9—C30	1.776 (3)	C11—H11B	0.9900
P1—O1	1.495 (2)	C11—C12	1.515 (4)
P1—N1	1.641 (3)	C12—H12A	0.9800
P1—N2	1.646 (3)	C12—H12B	0.9800
P1—N3	1.639 (3)	C12—H12C	0.9800
P2—O3	1.501 (2)	C13—H13A	0.9900
P2—N4	1.628 (3)	C13—H13B	0.9900
P2—N5	1.663 (2)	C13—C14	1.519 (5)
P2—N6	1.641 (2)	C14—H14A	0.9800
P3—O5	1.501 (2)	C14—H14B	0.9800
P3—N7	1.638 (3)	C14—H14C	0.9800
P3—N8	1.642 (3)	C15—H15A	0.9900
P3—N9	1.640 (2)	C15—H15B	0.9900
Na1—O2	2.393 (2)	C15—C16	1.516 (4)
Na1—O4	2.378 (2)	C16—H16A	0.9800
Na1—O6	2.333 (2)	C16—H16B	0.9800
Na1—O7	2.276 (2)	C16—H16C	0.9800
O2—C9	1.264 (3)	C17—H17A	0.9900
O4—C19	1.262 (3)	C17—H17B	0.9900
O6—C29	1.254 (4)	C17—C18	1.508 (5)
O7—H7A	0.86 (4)	C18—H18A	0.9800
O7—H7B	0.80 (4)	C18—H18B	0.9800
N1—C1	1.459 (4)	C18—H18C	0.9800
N1—C3	1.472 (4)	C19—C20	1.570 (4)
N2—C5	1.472 (4)	C21—H21A	0.9900
N2—C7	1.461 (4)	C21—H21B	0.9900
N3—C9	1.288 (3)	C21—C22	1.504 (5)
N4—C11	1.467 (3)	C22—H22A	0.9800
N4—C13	1.469 (4)	C22—H22B	0.9800
N5—C15	1.468 (4)	C22—H22C	0.9800
N5—C17	1.469 (4)	C23—H23A	0.9900
N6—C19	1.286 (3)	C23—H23B	0.9900
N7—C25	1.472 (4)	C23—C24	1.517 (5)
N7—C27	1.461 (4)	C24—H24A	0.9800
N8—C21	1.471 (4)	C24—H24B	0.9800
N8—C23	1.471 (4)	C24—H24C	0.9800
N9—C29	1.289 (4)	C25—H25A	0.9900
C1—H1A	0.9900	C25—H25B	0.9900
C1—H1B	0.9900	C25—C26	1.516 (4)
C1—C2	1.502 (5)	C26—H26A	0.9800
C2—H2A	0.9800	C26—H26B	0.9800
C2—H2B	0.9800	C26—H26C	0.9800
C2—H2C	0.9800	C27—H27A	0.9900
C3—H3A	0.9900	C27—H27B	0.9900
C3—H3B	0.9900	C27—C28	1.519 (5)
C3—C4	1.518 (5)	C28—H28A	0.9800
C4—H4A	0.9800	C28—H28B	0.9800
C4—H4B	0.9800	C28—H28C	0.9800
C4—H4C	0.9800	C29—C30	1.562 (4)
			
O1—Ca1—O2	79.33 (7)	H8B—C8—H8C	109.5
O1—Ca1—O3	94.32 (7)	O2—C9—N3	132.7 (3)
O1—Ca1—O4	148.96 (8)	O2—C9—C10	113.6 (2)
O1—Ca1—O5	94.39 (7)	N3—C9—C10	113.5 (2)
O1—Ca1—O6	118.09 (8)	Cl1—C10—Cl2	108.53 (17)
O2—Ca1—O4	77.47 (7)	Cl3—C10—Cl1	108.61 (19)
O3—Ca1—O2	119.61 (8)	Cl3—C10—Cl2	108.37 (18)
O3—Ca1—O4	79.71 (7)	C9—C10—Cl1	106.5 (2)
O3—Ca1—O5	94.12 (7)	C9—C10—Cl2	110.9 (2)
O3—Ca1—O6	146.96 (7)	C9—C10—Cl3	113.8 (2)
O5—Ca1—O2	145.92 (8)	N4—C11—H11A	108.9
O5—Ca1—O4	116.31 (7)	N4—C11—H11B	108.9
O5—Ca1—O6	78.07 (7)	N4—C11—C12	113.4 (3)
O6—Ca1—O2	75.74 (7)	H11A—C11—H11B	107.7
O6—Ca1—O4	75.45 (7)	C12—C11—H11A	108.9
O1—P1—N1	108.05 (13)	C12—C11—H11B	108.9
O1—P1—N2	115.74 (13)	C11—C12—H12A	109.5
O1—P1—N3	116.44 (12)	C11—C12—H12B	109.5
N1—P1—N2	104.75 (13)	C11—C12—H12C	109.5
N3—P1—N1	110.21 (14)	H12A—C12—H12B	109.5
N3—P1—N2	100.95 (13)	H12A—C12—H12C	109.5
O3—P2—N4	113.64 (12)	H12B—C12—H12C	109.5
O3—P2—N5	107.07 (12)	N4—C13—H13A	109.0
O3—P2—N6	116.65 (12)	N4—C13—H13B	109.0
N4—P2—Ca1	114.86 (9)	N4—C13—C14	113.1 (3)
N4—P2—N5	107.44 (13)	H13A—C13—H13B	107.8
N4—P2—N6	104.66 (12)	C14—C13—H13A	109.0
N6—P2—N5	106.89 (12)	C14—C13—H13B	109.0
O5—P3—N7	107.79 (12)	C13—C14—H14A	109.5
O5—P3—N8	115.28 (13)	C13—C14—H14B	109.5
O5—P3—N9	116.73 (12)	C13—C14—H14C	109.5
N7—P3—Ca1	128.31 (10)	H14A—C14—H14B	109.5
N7—P3—N8	106.03 (14)	H14A—C14—H14C	109.5
N7—P3—N9	107.54 (13)	H14B—C14—H14C	109.5
N9—P3—N8	102.78 (13)	N5—C15—H15A	109.2
O4—Na1—O2	77.48 (7)	N5—C15—H15B	109.2
O6—Na1—O2	76.19 (8)	N5—C15—C16	111.9 (2)
O6—Na1—O4	76.42 (8)	H15A—C15—H15B	107.9
O7—Na1—O2	141.26 (9)	C16—C15—H15A	109.2
O7—Na1—O4	134.11 (9)	C16—C15—H15B	109.2
O7—Na1—O6	126.09 (10)	C15—C16—H16A	109.5
P1—O1—Ca1	132.87 (11)	C15—C16—H16B	109.5
Ca1—O2—Na1	88.21 (7)	C15—C16—H16C	109.5
C9—O2—Ca1	131.21 (17)	H16A—C16—H16B	109.5
C9—O2—Na1	136.08 (18)	H16A—C16—H16C	109.5
P2—O3—Ca1	130.74 (10)	H16B—C16—H16C	109.5
Na1—O4—Ca1	88.29 (7)	N5—C17—H17A	108.5
C19—O4—Ca1	129.28 (16)	N5—C17—H17B	108.5
C19—O4—Na1	142.40 (17)	N5—C17—C18	114.9 (3)
P3—O5—Ca1	131.58 (11)	H17A—C17—H17B	107.5
Na1—O6—Ca1	89.86 (8)	C18—C17—H17A	108.5
C29—O6—Ca1	132.10 (17)	C18—C17—H17B	108.5
C29—O6—Na1	138.03 (18)	C17—C18—H18A	109.5
Na1—O7—H7A	120 (2)	C17—C18—H18B	109.5
Na1—O7—H7B	128 (3)	C17—C18—H18C	109.5
H7A—O7—H7B	111 (4)	H18A—C18—H18B	109.5
C1—N1—P1	119.8 (2)	H18A—C18—H18C	109.5
C1—N1—C3	116.4 (3)	H18B—C18—H18C	109.5
C3—N1—P1	118.9 (2)	O4—C19—N6	131.8 (2)
C5—N2—P1	117.6 (2)	O4—C19—C20	115.9 (2)
C7—N2—P1	121.1 (2)	N6—C19—C20	112.2 (2)
C7—N2—C5	115.1 (3)	Cl4—C20—Cl5	109.11 (16)
C9—N3—P1	125.5 (2)	Cl4—C20—Cl6	107.91 (15)
C11—N4—P2	119.8 (2)	Cl6—C20—Cl5	108.57 (16)
C11—N4—C13	115.9 (2)	C19—C20—Cl4	108.6 (2)
C13—N4—P2	123.43 (19)	C19—C20—Cl5	110.13 (19)
C15—N5—P2	116.78 (18)	C19—C20—Cl6	112.42 (19)
C15—N5—C17	114.6 (2)	N8—C21—H21A	109.0
C17—N5—P2	117.2 (2)	N8—C21—H21B	109.0
C19—N6—P2	124.6 (2)	N8—C21—C22	113.0 (3)
C25—N7—P3	123.4 (2)	H21A—C21—H21B	107.8
C27—N7—P3	119.6 (2)	C22—C21—H21A	109.0
C27—N7—C25	116.5 (2)	C22—C21—H21B	109.0
C21—N8—P3	118.0 (2)	C21—C22—H22A	109.5
C23—N8—P3	123.4 (2)	C21—C22—H22B	109.5
C23—N8—C21	116.2 (3)	C21—C22—H22C	109.5
C29—N9—P3	124.1 (2)	H22A—C22—H22B	109.5
N1—C1—H1A	108.6	H22A—C22—H22C	109.5
N1—C1—H1B	108.6	H22B—C22—H22C	109.5
N1—C1—C2	114.5 (3)	N8—C23—H23A	108.9
H1A—C1—H1B	107.6	N8—C23—H23B	108.9
C2—C1—H1A	108.6	N8—C23—C24	113.4 (3)
C2—C1—H1B	108.6	H23A—C23—H23B	107.7
C1—C2—H2A	109.5	C24—C23—H23A	108.9
C1—C2—H2B	109.5	C24—C23—H23B	108.9
C1—C2—H2C	109.5	C23—C24—H24A	109.5
H2A—C2—H2B	109.5	C23—C24—H24B	109.5
H2A—C2—H2C	109.5	C23—C24—H24C	109.5
H2B—C2—H2C	109.5	H24A—C24—H24B	109.5
N1—C3—H3A	109.5	H24A—C24—H24C	109.5
N1—C3—H3B	109.5	H24B—C24—H24C	109.5
N1—C3—C4	110.9 (3)	N7—C25—H25A	109.1
H3A—C3—H3B	108.1	N7—C25—H25B	109.1
C4—C3—H3A	109.5	N7—C25—C26	112.3 (3)
C4—C3—H3B	109.5	H25A—C25—H25B	107.9
C3—C4—H4A	109.5	C26—C25—H25A	109.1
C3—C4—H4B	109.5	C26—C25—H25B	109.1
C3—C4—H4C	109.5	C25—C26—H26A	109.5
H4A—C4—H4B	109.5	C25—C26—H26B	109.5
H4A—C4—H4C	109.5	C25—C26—H26C	109.5
H4B—C4—H4C	109.5	H26A—C26—H26B	109.5
N2—C5—H5A	109.1	H26A—C26—H26C	109.5
N2—C5—H5B	109.1	H26B—C26—H26C	109.5
N2—C5—C6	112.5 (3)	N7—C27—H27A	108.8
H5A—C5—H5B	107.8	N7—C27—H27B	108.8
C6—C5—H5A	109.1	N7—C27—C28	113.9 (3)
C6—C5—H5B	109.1	H27A—C27—H27B	107.7
C5—C6—H6A	109.5	C28—C27—H27A	108.8
C5—C6—H6B	109.5	C28—C27—H27B	108.8
C5—C6—H6C	109.5	C27—C28—H28A	109.5
H6A—C6—H6B	109.5	C27—C28—H28B	109.5
H6A—C6—H6C	109.5	C27—C28—H28C	109.5
H6B—C6—H6C	109.5	H28A—C28—H28B	109.5
N2—C7—H7C	108.7	H28A—C28—H28C	109.5
N2—C7—H7D	108.7	H28B—C28—H28C	109.5
N2—C7—C8	114.2 (3)	O6—C29—N9	131.7 (3)
H7C—C7—H7D	107.6	O6—C29—C30	113.9 (2)
C8—C7—H7C	108.7	N9—C29—C30	114.2 (3)
C8—C7—H7D	108.7	Cl8—C30—Cl7	109.84 (18)
C7—C8—H8A	109.5	Cl8—C30—Cl9	108.36 (17)
C7—C8—H8B	109.5	Cl9—C30—Cl7	107.63 (15)
C7—C8—H8C	109.5	C29—C30—Cl7	106.1 (2)
H8A—C8—H8B	109.5	C29—C30—Cl8	113.6 (2)
H8A—C8—H8C	109.5	C29—C30—Cl9	111.1 (2)
			
Ca1—P1—N1—C1	−6.1 (3)	O4—C19—C20—Cl6	5.3 (3)
Ca1—P1—N1—C3	148.2 (2)	O5—P3—N7—C25	−6.4 (3)
Ca1—P1—N2—C5	83.5 (3)	O5—P3—N7—C27	−177.6 (2)
Ca1—P1—N2—C7	−67.6 (3)	O5—P3—N8—C21	−56.9 (3)
Ca1—P1—N3—C9	0.5 (3)	O5—P3—N8—C23	104.9 (3)
Ca1—P2—N4—C11	−73.2 (2)	O5—P3—N9—C29	−20.8 (3)
Ca1—P2—N4—C13	95.4 (2)	O6—C29—C30—Cl7	−80.1 (3)
Ca1—P2—N5—C15	−159.31 (17)	O6—C29—C30—Cl8	159.1 (2)
Ca1—P2—N5—C17	−17.7 (3)	O6—C29—C30—Cl9	36.6 (3)
Ca1—P2—N6—C19	−12.7 (3)	N1—P1—O1—Ca1	−106.24 (19)
Ca1—P3—N7—C25	18.3 (3)	N1—P1—N2—C5	−62.6 (3)
Ca1—P3—N7—C27	−152.95 (19)	N1—P1—N2—C7	146.3 (3)
Ca1—P3—N8—C21	−90.1 (2)	N1—P1—N3—C9	115.3 (3)
Ca1—P3—N8—C23	71.6 (3)	N2—P1—O1—Ca1	136.72 (17)
Ca1—P3—N9—C29	−12.2 (2)	N2—P1—N1—C1	147.9 (2)
Ca1—O2—C9—N3	3.1 (5)	N2—P1—N1—C3	−57.8 (3)
Ca1—O2—C9—C10	−172.3 (2)	N2—P1—N3—C9	−134.3 (3)
Ca1—O4—C19—N6	25.0 (5)	N3—P1—O1—Ca1	18.4 (2)
Ca1—O4—C19—C20	−154.88 (18)	N3—P1—N1—C1	−104.2 (2)
Ca1—O6—C29—N9	23.4 (5)	N3—P1—N1—C3	50.1 (3)
Ca1—O6—C29—C30	−160.67 (18)	N3—P1—N2—C5	−177.1 (2)
P1—N1—C1—C2	86.6 (3)	N3—P1—N2—C7	31.8 (3)
P1—N1—C3—C4	144.0 (3)	N3—C9—C10—Cl1	−98.5 (3)
P1—N2—C5—C6	132.7 (3)	N3—C9—C10—Cl2	143.6 (2)
P1—N2—C7—C8	70.5 (4)	N3—C9—C10—Cl3	21.1 (4)
P1—N3—C9—O2	−2.2 (5)	N4—P2—O3—Ca1	−99.20 (16)
P1—N3—C9—C10	173.2 (2)	N4—P2—N5—C15	55.0 (2)
P2—N4—C11—C12	−116.5 (3)	N4—P2—N5—C17	−163.4 (2)
P2—N4—C13—C14	−89.5 (3)	N4—P2—N6—C19	102.7 (3)
P2—N5—C15—C16	−156.2 (2)	N5—P2—O3—Ca1	142.33 (15)
P2—N5—C17—C18	−81.3 (3)	N5—P2—N4—C11	78.0 (2)
P2—N6—C19—O4	−0.5 (5)	N5—P2—N4—C13	−113.4 (2)
P2—N6—C19—C20	179.5 (2)	N5—P2—N6—C19	−143.5 (3)
P3—N7—C25—C26	106.1 (3)	N6—P2—O3—Ca1	22.7 (2)
P3—N7—C27—C28	98.7 (3)	N6—P2—N4—C11	−168.6 (2)
P3—N8—C21—C22	−121.7 (3)	N6—P2—N4—C13	0.0 (3)
P3—N8—C23—C24	−81.7 (4)	N6—P2—N5—C15	−56.9 (2)
P3—N9—C29—O6	0.7 (5)	N6—P2—N5—C17	84.7 (2)
P3—N9—C29—C30	−175.2 (2)	N6—C19—C20—Cl4	66.0 (3)
Na1—Cl2—C10—Cl1	−90.41 (15)	N6—C19—C20—Cl5	−53.4 (3)
Na1—Cl2—C10—Cl3	151.81 (14)	N6—C19—C20—Cl6	−174.6 (2)
Na1—Cl2—C10—C9	26.2 (2)	N7—P3—O5—Ca1	138.69 (15)
Na1—Cl6—C20—Cl4	103.00 (13)	N7—P3—N8—C21	62.3 (3)
Na1—Cl6—C20—Cl5	−138.86 (12)	N7—P3—N8—C23	−136.0 (3)
Na1—Cl6—C20—C19	−16.8 (2)	N7—P3—N9—C29	−142.0 (2)
Na1—Cl9—C30—Cl7	68.32 (14)	N8—P3—O5—Ca1	−103.14 (17)
Na1—Cl9—C30—Cl8	−172.95 (13)	N8—P3—N7—C25	−130.3 (2)
Na1—Cl9—C30—C29	−47.42 (19)	N8—P3—N7—C27	58.4 (3)
Na1—O2—C9—N3	−145.1 (3)	N8—P3—N9—C29	106.4 (3)
Na1—O2—C9—C10	39.5 (4)	N9—P3—O5—Ca1	17.6 (2)
Na1—O4—C19—N6	−157.6 (2)	N9—P3—N7—C25	120.3 (2)
Na1—O4—C19—C20	22.5 (4)	N9—P3—N7—C27	−51.0 (3)
Na1—O6—C29—N9	−157.8 (2)	N9—P3—N8—C21	175.0 (2)
Na1—O6—C29—C30	18.1 (4)	N9—P3—N8—C23	−23.2 (3)
O1—P1—N1—C1	24.0 (3)	N9—C29—C30—Cl7	96.6 (3)
O1—P1—N1—C3	178.3 (2)	N9—C29—C30—Cl8	−24.2 (3)
O1—P1—N2—C5	56.2 (3)	N9—C29—C30—Cl9	−146.8 (2)
O1—P1—N2—C7	−94.8 (3)	C1—N1—C3—C4	−60.9 (4)
O1—P1—N3—C9	−8.2 (3)	C3—N1—C1—C2	−68.4 (4)
O2—C9—C10—Cl1	77.8 (3)	C5—N2—C7—C8	−81.3 (4)
O2—C9—C10—Cl2	−40.1 (3)	C7—N2—C5—C6	−74.6 (4)
O2—C9—C10—Cl3	−162.6 (2)	C11—N4—C13—C14	79.5 (3)
O3—P2—N4—C11	−40.2 (2)	C13—N4—C11—C12	74.1 (3)
O3—P2—N4—C13	128.4 (2)	C15—N5—C17—C18	61.1 (4)
O3—P2—N5—C15	177.4 (2)	C17—N5—C15—C16	61.3 (3)
O3—P2—N5—C17	−41.0 (2)	C21—N8—C23—C24	80.4 (4)
O3—P2—N6—C19	−23.8 (3)	C23—N8—C21—C22	75.2 (4)
O4—C19—C20—Cl4	−114.1 (2)	C25—N7—C27—C28	−73.1 (3)
O4—C19—C20—Cl5	126.5 (2)	C27—N7—C25—C26	−82.4 (4)

### Hydrogen-bond geometry (Å, º)

**Table:**

*D*—H···*A*	*D*—H	H···*A*	*D*···*A*	*D*—H···*A*
O7—H7*A*···O3^i^	0.86 (4)	2.23 (4)	2.959 (3)	143 (3)
O7—H7*B*···O5^i^	0.80 (4)	2.08 (4)	2.843 (3)	159 (4)

Symmetry code: (i) *x*, −*y*+1/2, *z*−1/2.
